# Genomic Evolution of the Increasing Prevalent Carbapenem‐Resistant Hypervirulent ST15 *Klebsiella pneumoniae*

**DOI:** 10.1155/ijm/8275904

**Published:** 2026-05-08

**Authors:** Shuaihua Fan, Pengcheng Du, Yunfei Tang, Huaiqing Qi, Chao Liu, Jun Guo

**Affiliations:** ^1^ Department of Pulmonary and Critical Care Medicine, Beijing Tsinghua Changgung Hospital, School of Clinical Medicine, Tsinghua Medicine, Tsinghua University, Beijing, China, tsinghua.edu.cn; ^2^ Medical Research Center, Beijing Institute of Respiratory Medicine and Beijing Chao-Yang Hospital, Capital Medical University, Beijing, China, ccmu.edu.cn; ^3^ Department of Infectious Disease, Peking University Third Hospital, Beijing, China, puh3.net.cn

**Keywords:** carbapenem resistance, hypervirulent, *Klebsiella pneumoniae*, ST15, virulence plasmid

## Abstract

**Purpose:**

ST15 *Klebsiella pneumoniae* (Kp) is increasingly prevalent in China and worldwide. Currently, the mechanisms underlying the acquisition of hypervirulence‐associated genes in sequence Type 15 (ST15) Kp are still not well studied. The global prevalence of ST15 Kp is increasing, alongside notable increases in both resistance and virulence.

**Methods:**

In this study, we collected 766 isolates of Kp from a tertiary hospital and 19,060 Kp genomes from the NCBI Assembly database. We analyzed the phylogenetic characteristics and profiles of hypervirulence‐associated genes among ST15 genomes, as well as their associations with other sequence types (STs).

**Results:**

We observed that the hypervirulence‐associated genes in the ST15 carbapenem‐resistant *Klebsiella pneumoniae* (CRKP) isolates were all located on plasmids, indicating the potential for horizontal transfer. Plasmid exchange and recombination are closely linked to transmission patterns in different regions. Notably, in Asia, lineages carrying the virulence plasmid genes *iucA* and *rmpA2* constitute the dominant population, accounting for 49.1% of the regional epidemic strains (with 48.5% lacking hypervirulence‐associated genes). The clustering of these isolates and their close genetic relationships suggest that hypervirulent ST15 strains are well adapted and spread rapidly in this region. Two main pathways for the acquisition of virulence plasmids by ST15 CRKP isolates were identified: One involves the recombination of nonvirulence plasmids with the classic virulence plasmid pVir from ST11, and the other involves the direct acquisition of virulence plasmids from other genera. We also found that one ST15 CRKP strain harbors multiple copies of key virulence genes.

**Conclusion:**

Overall, the genetic diversity of hypervirulence‐associated genes within ST15 CRKP isolates may enhance the ability to survive and spread globally, offering crucial evidence for clinical strategies aimed at managing this strain.

## 1. Introduction

*Klebsiella pneumoniae* (Kp) is an important opportunistic pathogen characterized by hypervirulence and multidrug resistance, which represent two distinct evolutionary pathways contributing to the global spread of high‐risk lineages. The increasing prevalence of antimicrobial resistance and its global spread, mediated by mobile genetic elements (MGEs), have made carbapenem‐resistant *Klebsiella pneumoniae* (CRKP) an urgent threat in healthcare settings, associated with high mortality [1, 2]. Since the 1980s, the number of Kp strains isolated from community‐acquired infections has gradually increased, primarily in healthy young individuals. These infections were eventually defined as hypervirulent *Klebsiella pneumoniae* (hvKp) [3]. The rapid progression and poor prognosis associated with hvKp infection have posed a significant challenge to public health [4]. Historically, multidrug resistance and hypervirulence were considered distinct phenotypes, as CRKP isolates typically exhibit relatively low virulence, whereas hvKp isolates are usually sensitive to multiple antimicrobial agents. However, an increasing number of Kp strains with convergence of both multidrug resistance and hypervirulence, designated hypervirulent carbapenem‐resistant *Klebsiella pneumoniae* (Hv‐CRKP), have been identified in recent years [5].

In addition to the high prevalence of the endemic clone ST11 in China, variants of the classical *Klebsiella pneumoniae* (cKp) ST15 with increased antimicrobial resistance genes have emerged. A multicenter prospective study in Vietnam revealed that the prevalence of ST15 was higher than that of ST11. Similar to ST1, ST15 also exhibited high levels of antimicrobial resistance [6, 7]. As an emerging high‐risk subtype, ST15 CRKP has become an endemic clone in hospital‐acquired infections and tends to acquire more hypervirulence‐associated genes [8]. However, the characteristics of ST15 Hv‐CRKP remain poorly understood.

Recent studies have shown that any combination of *iroB*, *iucA*, *peg-344*, *rmpA*, or *rmpA2* serves as a key determinant of hvKp phenotype [9]. The virulence plasmid (pVir) pLVPK was previously identified as the main contributor to hypervirulence, particularly within classical hvKp clones [10, 11]. Meanwhile, epidemic lineages of ST11 CRKP evolved into Hv‐CRKP through the acquisition of pLVPK‐like pVirs [5, 12]. A conjugative pVir in *Klebsiella variicola* was formed by integrating fragments of the pLVPK plasmid into a transferable IncFIB‐type plasmid [13]. These findings highlight the diversity of plasmids involved in the convergence of resistance and virulence.

ST15, a classical cKp subtype, tends to acquire more hypervirulence‐associated genes and is transitioning into Hv‐CRKP. Therefore, in this study, we conducted a large‐scale genomic epidemiological analysis to explore the evolution of ST15 Hv‐CRKP. Our findings highlight the role of MGE‐mediated transfer of hypervirulence‐associated genes in the emergence of ST15 Hv‐CRKP and suggest that multiple mechanisms may be involved in the formation of pVirs in ST15.

## 2. Materials and Methods

### 2.1. Genomic Data and Background Information

We collected 766 Kp isolates from a tertiary hospital, with all isolates meeting the inclusion criteria of being obtained from patients aged 18 years or older and confirmed as Kp infections. Species identification was initially performed using a Bruker matrix‐assisted laser desorption/ionization time‐of‐flight (MALDI‐TOF) mass spectrometer at Beijing Tsinghua Changgung Hospital. All isolates were obtained from clinical specimens of inpatients and outpatients, with sample collection performed by nurses following standard clinical sampling protocols and were confirmed as Kp isolates by the clinical laboratory. Antimicrobial susceptibility testing was subsequently conducted with the Mérieux Vitek Compact II system, and all isolates were preserved at −80°C for subsequent analysis.

Additionally, to infer global epidemiological trends and provide broader context, we retrieved 19,060 Kp genomes with corresponding metadata from the NCBI Assembly database (database closed March 2024). After performing quality control and selecting data based on completeness of background information and sequencing data quality, we combined these publicly available global data with our local dataset for comparative analysis. Our data served as a supplementary cohort to enhance the representativeness of the overall analysis. Among these, 14,728 (77.3%) genome sequences derived from clinical isolates, providing information on the country and year of isolation, were enrolled in the study. Categorized by genome assembly level, this dataset can be divided into four subgroups: complete genome (*n* = 1296), chromosome (*n* = 121), scaffold (*n* = 4276), and contig (*n* = 9,035). The 1417 genomes had complete assemblies, with sizes ranging from 5 to 7 Mbp, and were identified as Kp through Kleborate [14].

### 2.2. Whole‐Genome Sequencing and Analysis

Whole genomic DNA was extracted by TIANamp bacterial DNA kit (Tiangen Biotech, Beijing, China) and sequenced on a NovaSeq 6000 platform using the Illumina method to obtain 150‐bp paired‐end reads. Low‐quality reads were filtered by fastp software (https://github.com/OpenGene/fastp). The cleaned data were de novo assembled by SPAdes v3.15.2. Gene prediction was performed with Prokka [15], whereas MGEs were further identified using the mobileOG‐db database [16]. EasyFig [17] was used to analyze the genetic background and homologous regions containing hypervirulence genes. The online interactive software Proksee (https://proksee.ca/) was employed to visualize the genomic circos plot. Furthermore, we used Roary [18] for pangenome analysis to construct a gene presence–absence table. We utilized the tidyverse package in R to create a gene sharing table.

### 2.3. Identification of Molecular Subtypes, Antimicrobial Resistance, and Virulence

The identification of STs, resistance genes and scores, and virulence genes and scores were performed by processing genomic sequences with Kleborate. CRKPs were defined as isolates with resistance score ≥ 2. The resistance scores were defined as follows: 0, without ESBL and carbapenemase; 1, positive for ESBL and no carbapenemase; 2, positive for carbapenemase without colistin resistance; and 3, positive for carbapenemase with colistin resistance [19]. HvKp is determined by any combination of the virulence genes *iucA*, *iroB*, *rmpA*, *rmpA2*, or *peg-344*. The *iucA* and *iroB* were identified using Kleborate, *rmpA* and *rmpA2* were identified using the VFDB [20] database, and *peg-344* was identified using BLAST 2.15.0+ [21] (requiring sequence identity ≥ 90*%* and coverage ≥ 80*%*).

### 2.4. Identification of pVirs

pVirs were defined as plasmids carrying at least one of the five key hypervirulence‐associated genes (*iucA*, *iroB*, *rmpA*, *rmpA2*, or *peg-344*). Plasmid incompatibility (Inc) types were determined using PlasmidFinder (v2.1) [22] with default thresholds (≥ 90% identity and ≥ 80% coverage). Plasmid clustering and phylogenetic relationships were inferred based on core gene alignment of plasmid sequences using Roary. Average nucleotide identity (ANI) among plasmids was calculated using fastANI (v1.33) [23] with a threshold of ≥ 95% to define related plasmids. Plasmids with low assembly quality (total length < 5 kb or > 20% ambiguous bases) were excluded from further analysis.

### 2.5. Phylogenetic Analysis

Genomic sequence alignment and single‐nucleotide polymorphism (SNP) calling were performed using Snippy v4.6.0 (https://github.com/tseemann/snippy), followed by recombination filtering of the core genome alignment with Gubbins (https://github.com/nickjcroucher/gubbins) to remove horizontally acquired regions. A maximum‐likelihood phylogenetic tree was then reconstructed from the recombination‐free SNP alignment using RAxML under the GTRGAMMA model with 1000 bootstrap replicates to infer robust evolutionary relationships among all ST15 genomes. Pairwise SNP distances were calculated to delineate genetic clusters and define phylogenetic branches. The resulting tree was visualized and annotated using iTOL (https://itol.embl.de/). Isolates were further classified into strain lineages according to the presence of hypervirulence‐associated genes, and a minimum spanning tree based on pairwise SNP distances was generated and visualized with PhyloViz [24] to illustrate population structure and genetic relatedness.

### 2.6. Statistical Analysis

We calculated the proportions of Hv‐CRKP and its subtypes. The gene sharing network graph was plotted using the Python modules pandas, networkx, matplotlib.pyplot, and matplotlib.patches.

## 3. Results

### 3.1. ST15 CRKP Acquired Hypervirulence Genes Through Horizontal Gene Transfer and Spread Globally

In the Kp genome dataset obtained from NCBI, Kleborate identified a total of 986 genomes of clonal Group 15 (CG15), including 974 ST15, 11 ST15‐1LV, and 1 ST15‐2LV. Among the clinical isolates from our hospital, there were 27 genomes of CG15, comprising 25 ST15 genomes and 2 ST15‐2LV genomes. Therefore, a total of 1013 CG15 genomes were included in the study.

**Figure 1:**
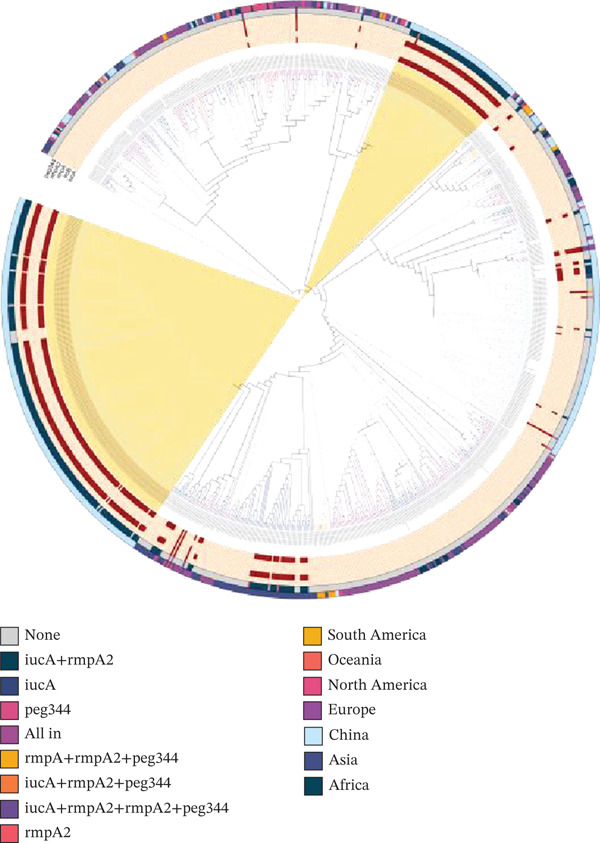
Phylogenetic tree of enrolled ST15 genomes. Two clades from China (Chinese Clades 1 and 2) exhibited significant clustering, with most isolates carrying the key virulence genes *iucA* and *rmpA2*. A total of 12,158 single‐nucleotide polymorphisms (SNPs) were identified across these two clades, of which 10,512 were classified as conserved, indicating the existence of two clonal groups.

Across all ST15 Kp strains, a total of 23,291 SNPs were identified and used to construct a phylogenetic tree (Figure 1). Notably, two clades primarily composed of isolates from China exhibited significant clustering, with most of them carrying the key virulence genes *iucA* and *rmpA2*. Compared with the reference genome, these two clades possessed 12,158 SNPs in total, of which 10,512 were shared by the two clades, indicating 1646 differences between them. This high proportion of shared SNPs suggests regional expansion of closely related clones. Our genomic analysis indicated that the genetic distance between these two phylogroups is relatively low, suggesting that they are evolutionarily close to each other.

**Figure 2:**
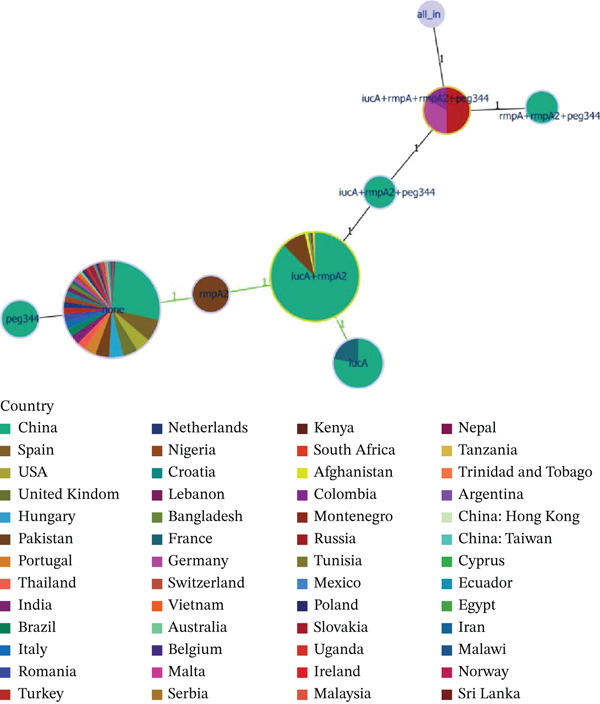
A minimum spanning tree using the virulence gene patterns of global ST15 Hv‐CRKP isolates. The length of the lines connecting each circle describes the difference in virulence genes between the two patterns, whereas the color of the fans represents different countries.

Virulence genes were all located on plasmids, and recombination events were observed frequently. The ST15 Hv‐CRKP isolates were categorized into nine groups according to different combinations of the five virulence genes (Table 1 and Figure 1). Among these groups, the “None” group represents classical CRKP isolates without any hypervirulence genes. The virulence gene profiles of ST15 Hv‐CRKP isolates were analyzed through clustering visualization (Figure 2). The most prevalent genotype, carrying both *iucA* and *rmpA2* genes, accounted for the highest proportion (31.9%, 323/1013) and was predominantly distributed among Asian isolates (98.1%, 317/323). This specific gene combination was also identified in five European isolates.

**Table 1:** Groups of ST15 Hv‐CRKP based on the carriage of virulence genes.

Group	Number
None	666
*i* *u* *c* *A* + *r* *m* *p* *A*2	323
*iucA*	9
*i* *u* *c* *A* + *r* *m* *p* *A* + *r* *m* *p* *A*2 + *p* *e* *g* − 344	6
*i* *u* *c* *A* + *i* *r* *o* *B* + *r* *m* *p* *A* + *r* *m* *p* *A*2 + *p* *e* *g* − 344	3
*peg-344*	2
*rmpA2*	2
*i* *u* *c* *A* + *r* *m* *p* *A*2 + *p* *e* *g* − 344	1
*r* *m* *p* *A* + *r* *m* *p* *A*2 + *p* *e* *g* − 344	1

### 3.2. Potential Acquisition of Virulence Genes in ST15 Hv‐CRKP From ST11 Strains

To further explore the sources of virulence genes in ST15, we analyzed the complete genomes of 1417 Kp isolates from NCBI, including 74 ST15 isolates, 17 of which were ST15 Hv‐CRKP. A total of 4803 plasmids were identified, with 10 low‐confidence plasmids excluded from further analysis based on criteria such as sequence quality, coverage, and alignment scores. Among the remaining plasmids, 381 were pVirs from Hv‐CRKP. These plasmids encoded 26,521 genes in total, 2248 of which were part of the plasmid core genome and were linked primarily to plasmid transfer and horizontal gene transfer. Gene presence–absence matrices and plasmid relationships were analyzed (Figure S1). The Kp isolates showed no distinct clustering, and the ST15 plasmids displayed significant dispersion without the formation of specific clusters.

**Figure 3:**
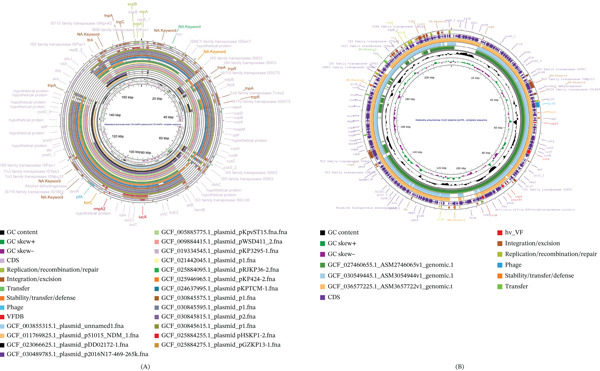
Comparison of regions containing virulence genes. (A) Comparison between ST15 plasmids containing only *iucA* and *rmpA2* and the classic virulence plasmid pVir from ST11 (pVir‐CR‐HvKp4 plasmid). (B) Comparison of the genomic between three ST15 Hv‐CRKP isolates carrying all five virulence genes and the classic virulence plasmid pLVPK from ST11.

ST11 tends to acquire pVir plasmids to evolve into hypervirulent isolates [25]. Given that both ST15 and ST11 are classical CRKP, and considering the distribution of the ST15 Hv‐CRKP group, we hypothesized that the plasmids carrying the *iucA* and *rmpA2* combination in ST15 strains may be related to pVir‐like plasmids associated with ST11. We screened 17 complete ST15 Hv‐CRKP genomes and identified 17 pVirs (Table S1). These plasmids were compared with the typical pVir pVir‐CR‐HvKp4 (Figure 3A). Most regions near *iucA* and *rmpA2* shared similar gene environments with pVir‐CR‐HvKp4.

In the phylogenetic tree, three ST15 Hv‐CRKP isolates carried all five virulence genes. Since the genomes of these three isolates are incomplete, we compared the genomes of these three ST15 Hv‐CRKP isolates with the CG43‐plasmid‐pLVPK plasmid (Figure 3B). The results revealed that these three isolates had over 70% coverage with the CG43‐plasmid‐pLVPK plasmid, indicating significant genetic similarity. Furthermore, the localization and arrangement of the hypervirulence genes were nearly identical, suggesting a shared evolutionary ancestry or horizontal exchange between lineages.

### 3.3. Two Ways in Which Hv‐CRKP ST15 Acquires Hypervirulence Genes

We compared 17 ST15 Hv‐CRKP plasmids with the classical pVir plasmid pVir‐CR‐HvKp4 (Figure 3A) and identified four distinct distribution patterns. Four representative isolates were selected for further analysis (Figures 4a–d and S2a–d). The Plasmids CGF_003855315.1 unnamed1, GCF_005885775.1 pKpvST15, and GCF_011769825.1 p51015 NDM_1 contained relatively large homologous segments with the pVir‐CR‐HvKp4 plasmid. Their virulence gene contexts were consistent with those of the pVir‐CR‐HvKp4 plasmid. Highly similar insertion sequence (IS) elements and other MGEs were observed near the *iucA* and *rmpA2* regions, with 100% identity and coverage.

Figure 4Collinearity analysis of the following plasmids with the pVir‐CR‐HvKp4 plasmid: (a) CGF_003855315.1 unnamed1, (b) GCF_005885775.1 pKpvST15, (c) GCF_011769825.1 p51015 NDM_1, and (d) GCF_025884255.1 pHSKP1‐2. Additionally, we performed (e) a collinearity analysis of GCF_025884275.1 pGZKP13‐1 with the pVir‐CR‐HvKp4 plasmid derived from GCF_025884255.1 pHSKP1‐2.

**Figure:**
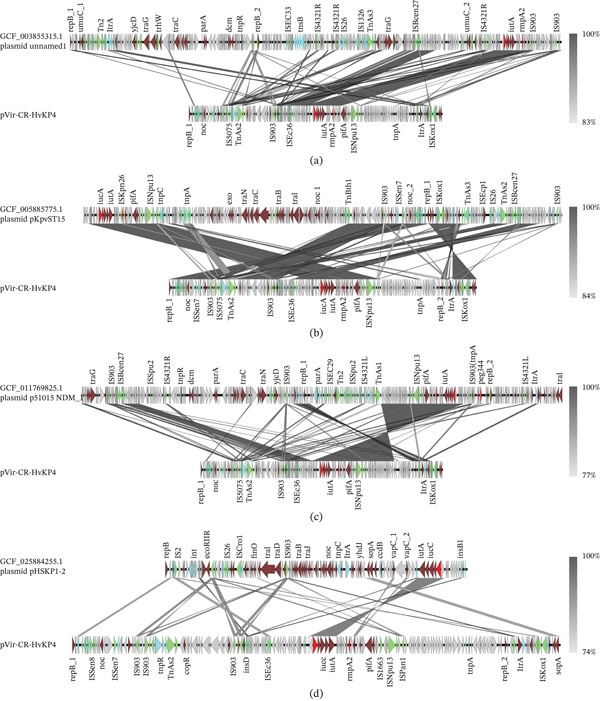
(A)

**Figure:**
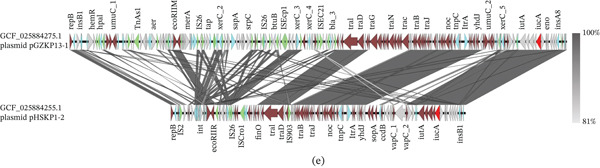
(B)

**Figure 5:**
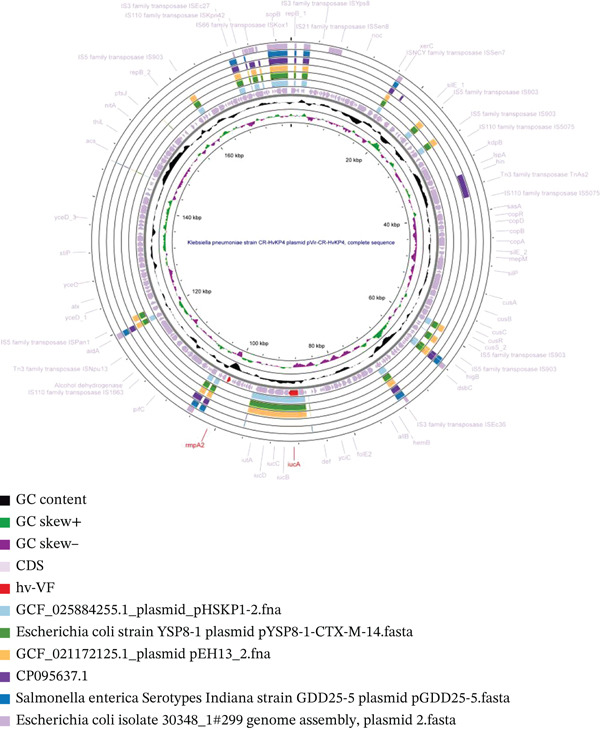
Circular comparison map of virulence plasmids. From the inside outwards, the first circle depicts the plasmid GCF_025884255.1 pHSKP1‐2 (Shanghai, China, 2018). The second and third circles correspond to two highly similar virulence plasmids, which are from the *Escherichia coli* strain YSP8‐1 plasmid pYSP8‐1‐CTX‐M‐14 (Guangdong, China, 2017) and the *Klebsiella pneumoniae* strain GCF_021172125.1 Plasmid pEH13_2 (Hong Kong, China, 2017, ST5605), respectively. The fourth to sixth circles represent three nonvirulence plasmids that have good alignment. Notably, there were no nonvirulence plasmids from Kp with high identity and coverage; most were derived from *E*. *coli*, with a relatively small number from *Salmonella enterica*.

Plasmid GCF_025884275.1 pGZKP13‐1 exhibits distinct characteristics compared with other identified pVirs. It is most similar to plasmid GCF_025884255.1 pHSKP1‐2, with both plasmids containing only the *iucA* gene. Sequence comparison showed high similarity between these two plasmids (Figures 4e and S2e). A BLAST search of the GCF_025884255.1 Plasmid pHSKP1‐2 in NCBI revealed high similarity with two pVirs carrying *iucA*: *Escherichia coli* pYSP8‐1‐CTX‐M‐14 (99.66% identity with 97% coverage) and the GCF_021172125.1 Plasmid pEH13_2 (99.66% identity with 97% coverage). It also showed high similarity with two non‐pVirs: *E*. *coli* 30348_1 Plasmid 2 (99.44% identity with 56% coverage) and *Salmonella enterica* pGDD25‐5 (99.51% identity with 71% coverage) (Figure 5).

**Figure 6:**
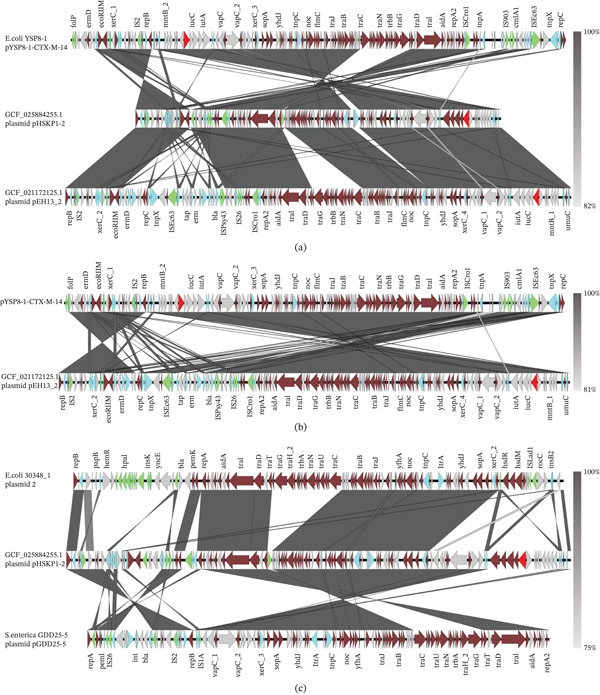
Collinearity map of plasmids. (a) The collinearity analysis of two virulence plasmids: pYSP8‐1‐CTX‐M‐14 and GCF_021172125.1 plasmid pEH13_2, compared with the ST15 GCF_025884255.1 Plasmid pHSKP1‐2. (b) The collinearity analysis of the same two virulence plasmids, pYSP8‐1‐CTX‐M‐14 and GCF_021172125.1 Plasmid pEH13_2. (c) The collinearity analysis of two nonvirulence plasmids: 30348_1 Plasmid 2 and pGDD25‐5, in relation to the ST15 GCF_025884255.1 Plasmid pHSKP1‐2.

Colinearity analysis of the aligned pVirs revealed that both pYSP8‐1‐CTX‐M‐14 and GCF_021172125.1 Plasmid pEH13_2 were highly similar to GCF_025884255.1 Plasmid pHSKP1‐2 (Figures 6a,b and S3a‐3b). A comparison between pYSP8‐1‐CTX‐M‐14 and GCF_021172125.1 Plasmid pEH13_2 showed that the two plasmids were nearly identical.

Collinearity analysis of the aligned non‐pVirs revealed that the unaligned regions corresponded to the locations of virulence genes (Figures 6c and S3c). No closely related isolates of these non‐pVirs were identified within the *Klebsiella* genus.

### 3.4. Amplification of Virulence Genes in an ST15 Strain

**Figure 7:**
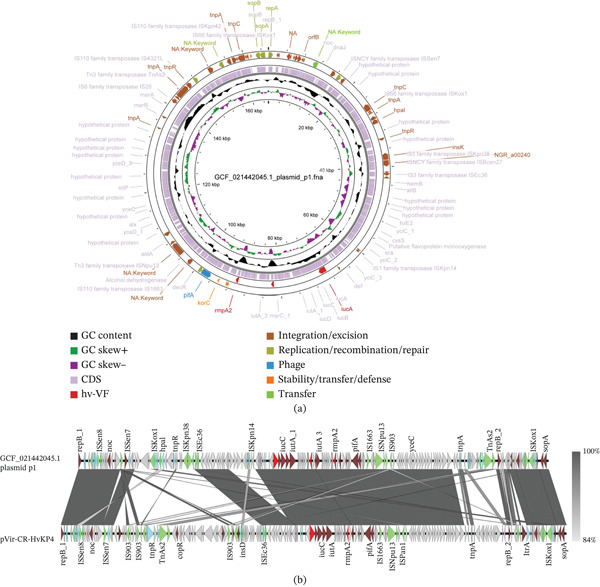
The genomic circular map (Panel (a)) illustrates the structure of plasmid GCF_021442045.1 (p1), whereas Panel (b) presents the collinearity analysis between this plasmid and pVir‐CR‐HvKp4. In the figures, red indicates high‐virulence genes, green represents IS elements, blue denotes transposons, dark red marks other functional genes, and gray marks gene regions encoding hypothetical proteins as predicted by Prokka. Notably, plasmid GCF_021442045.1 was found to contain two copies of the *rmpA2* gene.

We also identified a distinctive ST15 Hv‐CRKP plasmid, designated as GCF_021442045.1 Plasmid p1 (Figure 7a). Analysis of this long‐read sequenced and fully assembled plasmid revealed multiple copies of virulence genes. Specifically, the *rmpA2* gene was observed at adjacent loci within the plasmid, exhibiting 100% nucleotide identity and over 90% sequence coverage between copies. Based on this complete plasmid assembly, which was derived from PacBio long‐read sequencing and is available in the public database, the observed duplication appears to represent a genuine structural feature rather than an assembly artifact. To our knowledge, this specific configuration has not been widely reported in previous studies.

Further analysis revealed that the distribution of virulence genes in GCF_021442045.1 Plasmid p1 is most similar to that of other ST15 Hv‐CRKP plasmids. To further explore the relationship between GCF_021442045.1 Plasmid p1 and other plasmids, we constructed a gene collinearity map and compared it with that of the pVir‐CR‐HvKp4 plasmid (Figures 7b and S4). The results revealed that GCF_021442045.1 Plasmid p1 shows high overall similarity to pVir‐CR‐HvKp4. Specifically, a long segment carrying virulence genes is completely identical.

Notably, we observed significant copy number variation in the region adjacent to the *rmpA2* gene in GCF_021442045.1 Plasmid p1. We speculate that this variation in gene structure may have been acquired through mechanisms such as horizontal gene transfer or intraplasmid recombination, indicating an evolutionary adaptation of this strain in terms of pathogenicity, which may increase its competitiveness in clinical infections.

## 4. Discussion

ST15 Kp is an emerging high‐risk clone that has become a significant public health threat, leading to the emergence of ST15 HvKP along with the spread of hybrid plasmids carrying both virulence and antibiotic resistance traits [26]. We analyzed the genomic characteristics of 19,060 Kp genomes from NCBI, together with 766 Kp genomes collected from a tertiary hospital in Beijing, to investigate the evolution of ST15 Hv‐CRKP. We found that a portion of ST15 Kp evolved into hypervirulent strains by acquiring virulence genes through horizontal gene transfer from ST11 Kp, whereas another portion formed hypervirulent strains by directly acquiring pVirs from other genera. Meanwhile, the presence of multiple copies of virulence genes in ST15 strains poses new challenges for the prevention of ST15 Hv‐CRKP.

Analysis of the genomes of 14,728 clinical isolates of Kp from NCBI revealed significant regional variation in the distribution of these isolates (Supporting Information). There has been an annual increase in both resistance and virulence scores, with a notable increase in CRKP since 2010. This trend further confirms the worsening problem of antibiotic resistance and virulence in Kp on a global scale. Additionally, we observed that, similar to ST11, ST15 clinical isolates have markedly increased since 2010 [6] (Supporting Information), particularly the number of hypervirulent, multidrug‐resistant isolates.

As an important subtype of CRKP, the evolution of hypervirulent isolates of ST15 is particularly prominent, especially in Asia (619/986, 62.8%), where ST15 Hv‐CRKP shows a monophyletic clustering trend in the phylogenetic tree. The minimum spanning tree revealed that the combination of *iucA* and *rmpA2* represented the most predominant lineage. The *iucA* gene encodes proteins involved in iron utilization, whereas *rmpA2* is closely associated with capsular polysaccharide synthesis. The co‐occurrence of these two genes enhances the virulence of the isolates, thereby increasing their survival and transmission capabilities in hosts.

All key virulence genes (*iucA*, *iroB*, *rmpA*, *rmpA2*, and *peg-344*) in the ST15 Hv‐CRKP genomes were located on plasmids. The clustering of pVirs from *Klebsiella* was dispersed, indicating that the plasmids of ST15 strains have multiple, distinct evolutionary lineages. This observation suggests that transfer events between the plasmids of ST15 and other sequences types occur frequently, resulting in the integration of genes from different sequence types into the ST15 plasmids, or that ST15 has acquired pVirs from multiple sources, leading to considerable diversity in its genetic characteristics. This mechanism is supported by evidence at the genetic level: Although the acquisition of *iuc5* from *E. coli* is well‐documented [27], our study identified multiple other *iuc* variants (including but not limited to types *iucA_1, iucA_45,* and *iucA_42*) that likely also originated from *E. coli*.

Gene colinearity analysis revealed a high degree of similarity between the pVirs of ST15 isolates and the classic pVir plasmid of ST11 isolates. Additional analyses also revealed high similarity between three ST15 isolates that carry a relatively large number of hypervirulence genes and the ST11 pLVPK plasmid. These findings suggest potential horizontal gene transfer events and imply that the hypervirulence genes in ST15 may have originated from the acquisition of the ST11 plasmid. We speculate that these genes may have been transferred to ST15 Hv‐CRKP isolates from ST11 isolates through mechanisms such as horizontal gene transfer or plasmid recombination. The existence of such gene transfer phenomena further reveals the ability of bacteria to adapt to new environments during evolution and may lead to the emergence of new pathogenic phenotypes.

Furthermore, we identified two pVirs that carry only the *iucA* gene and show low similarity to the pVir plasmid, consisting of short, similar fragments. Further analysis revealed that these plasmids exhibit high similarity and coverage with pVirs from other genera, whereas non‐pVirs with high similarity and coverage were not found within the *Klebsiella* genus. This is consistent with the existence of a cross‐genus acquisition pathway for pVirs. Importantly, this type of pVir, acquired from other genera, may render ST15 Kp a more threatening pathogen, posing new challenges for clinical treatment and emphasizing the necessity for monitoring and preventive measures against similar cross‐genus transmission events.

Our study also identified a unique ST15 strain that carries multiple copies of the *rmpA2* gene, highlighting the diversity and complexity of its plasmid genomes. This observation suggests that gene duplication and horizontal gene transfer may be important mechanisms through which Kp adapts to different environments and increases its pathogenicity. The amplification of virulence genes such as *rmpA2* may increase the production of capsular polysaccharides, thereby improving the ability of bacteria to evade the host immune response and establish successful infections. This finding emphasizes the importance of understanding the genetic mechanisms that contribute to the adaptability and virulence of ST15 isolates, as well as the potential implications for treatment strategies in clinical settings.

This study relies primarily on genomic data from public databases and a single‐center sample collection, which, despite its scale, carries inherent methodological limitations. Bioinformatics analyses depend on reference‐based alignment and database annotations, lack experimental validation, and the static nature of genomic snapshots restricts our ability to fully reconstruct the dynamic spatiotemporal processes of plasmid transfer and recombination in real‐world settings. Therefore, future research should expand clinical sampling and conduct long‐term follow‐up to elucidate the pathways through which ST15 CRKP evolves and acquires hypervirulence genes in different clinical environments. Furthermore, given the complexity of horizontal plasmid transfer, studies should integrate plasmid recombination breakpoint analysis with time‐calibrated phylogeny to trace transmission directions, as well as employ experimental approaches such as genome editing techniques and in vivo models to delineate the molecular mechanisms of plasmid dissemination. This study enhances the understanding of the global spread of ST15 Hv‐CRKP and highlights the need to establish more effective surveillance and intervention measures to curb the evolving epidemic of CRKP.

## 5. Conclusion

This study elucidates two potential pathways for hypervirulence acquisition in ST15 CRKP: recombination with pVir‐like pVirs from ST11 strains and direct inter‐genus acquisition of novel pVirs. The high prevalence of the *i* *u* *c* *A* + *r* *m* *p* *A*2 clone in Asia (317/323, 98.1%), along with observed instances of virulence gene amplification, may indicate enhanced fitness and transmission potential in this lineage. Collectively, these genomic findings provide a basis for further investigation and inform the development of surveillance strategies targeting this emerging public health concern.

## Author Contributions

J.G. and C.L. conceived the study and acquired funding; Y.T. and H.Q. collected the data; S.F. conducted bioinformatics analysis; Y.T. and H.Q. performed clinical data analysis; S.F. and P.D. drafted the manuscript; P.D., J.G., and C.L. supervised the research and edited the manuscript. S.F. and P.D. contributed equally to this article and share the position of first author.

## Funding

This work was supported by the National Natural Science Foundation of China (Nos. 82200012 and 82470010) (Dr. Liu), Peking University Clinical Scientist Training Program (BMU2024PYJH011) (Dr. Liu), and the Fundamental Research Funds for the Central Universities (Dr. Liu), and High‐level Public Health Technical Talents Construction Project Training Program of Beijing Municipal Health Commission (Discipline Leader, No. 02‐06) (Dr. Guo).

## Ethics Statement

This study was approved by the Ethics Committee of Beijing Tsinghua Changgung Hospital, with the Ethical Approval Number 18116‐0‐01, and the Guidelines for Human Experimentation (PRC) were followed throughout. Informed consent was not obtained for this study since clinical data were de‐identified.

## Consent

The authors have nothing to report.

## Conflicts of Interest

The authors declare no conflicts of interest.

## Supporting Information

Additional supporting information can be found online in the Supporting Information section.

## Supporting information


**Supporting Information 1** Figure S1: Gene sharing network of all (a) plasmids, and (b, c) highly virulent plasmids. The only distinction between (b) and (c) is the color of the interaction lines.

**Supporting Information 2** Figure S2: Detailed genomic locations of Figure 4: Collinearity analysis of the following plasmids with the pVir‐CR‐HvKp4 plasmid: (a) plasmid unnamed1 from Strain KP_NORM_BLD_2014_104014 (Accession Number CGF_003855315.1), (b) pKpvST15 from Strain KpvST15_NDM (Accession Number GCF_005885775.1), (c) p51015 NDM_1 from Strain 51015 (Accession Number GCF_011769825.1), and (d) Plasmid pHSKP1‐2 from Strain HSKP1 (Accession Number GCF_025884255.1). Additionally, we performed (e) a collinearity analysis between Plasmid pGZKP13‐1 (from Strain GZKP13; Accession Number GCF_025884275.1) and the pVir‐CR‐HvKp4 plasmid (derived from pHSKP1‐2).

**Supporting Information 3** Figure S3. Detailed genomic locations of Figure 6. Panel (a) shows the collinearity analysis of two virulence plasmids: pYSP8‐1‐CTX‐M‐14 and Plasmid pEH13_2 (GCF_021172125.1), compared with the ST15 Plasmid pHSKP1‐2 (GCF_025884255.1). Panel (b) focuses on the collinearity analysis of the same two virulence plasmids, pYSP8‐1‐CTX‐M‐14 and Plasmid pEH13_2. Panel (c) examines the collinearity analysis of two nonvirulence plasmids: 30348_1 Plasmid 2 and pGDD25‐5, in relation to the ST15 Plasmid pHSKP1‐2.

**Supporting Information 4** Figure S4. Detailed genomic locations of Figure 7b. Collinearity analysis between plasmid p1 and pVir‐CR‐HvKp4.

**Supporting Information 5** Table S1: This table provides a detailed breakdown of the virulence gene repertoire for all 17 ST15 hv‐CRKP isolates analyzed in this study, listing the specific loci present in each isolate.

**Supporting Information 6** This Supporting Information analyzes temporal trends in the resistance and virulence of clinical *K. pneumoniae*, revealing a significant global increase in CRKP since 2010.

## Data Availability

The data are provided by National Microbiology Data Center. The URL is https://nmdc.cn/resource/genomics/project/detail/NMDC10019542.

## References

[1] Huang W. , Qiao F. , Zhang Y. , Huang J. , Deng Y. , Li J. , and Zong Z. , In-Hospital Medical Costs of Infections Caused by Carbapenem-Resistant *Klebsiella pneumoniae* , Clinical Infectious Diseases. (2018) 67, no. supplement 2, S225–S230, 10.1093/cid/ciy642, 2-s2.0-85056509232, 30423052. 30423052

[2] Nordmann P. , Cuzon G. , and Naas T. , The Real Threat of *Klebsiella pneumoniae* Carbapenemase-Producing Bacteria, Lancet infectious diseases. (2009) 9, no. 4, 228–236, 10.1016/S1473-3099(09)70054-4, 2-s2.0-62749195559, 19324295. 19324295

[3] Shon A. S. , Bajwa R. P. , and Russo T. A. , Hypervirulent (Hypermucoviscous) *Klebsiella pneumoniae*: A New and Dangerous Breed, Virulence. (2013) 4, no. 2, 107–118, 10.4161/viru.22718, 2-s2.0-84874209547, 23302790. 23302790 PMC3654609

[4] Lan P. , Jiang Y. , Zhou J. , and Yu Y. , A Global Perspective on the Convergence of Hypervirulence and Carbapenem Resistance in *Klebsiella pneumoniae* , Journal of Global Antimicrobial Resistance. (2021) 25, 26–34, 10.1016/j.jgar.2021.02.020, 33667703. 33667703

[5] Gu D. , Dong N. , Zheng Z. , Lin D. , Huang M. , Wang L. , Chan E. W. C. , Shu L. , Yu J. , Zhang R. , and Chen S. , A Fatal Outbreak of ST11 Carbapenem-Resistant Hypervirulent *Klebsiella pneumoniae* in a Chinese Hospital: A Molecular Epidemiological Study, Lancet Infectious Diseases. (2018) 18, no. 1, 37–46, 10.1016/S1473-3099(17)30489-9, 2-s2.0-85028557993, 28864030. 28864030

[6] Chen J. , Hu C. , Wang R. , Li F. , Sun G. , Yang M. , and Chu Y. , Shift in the Dominant Sequence Type of Carbapenem-Resistant *Klebsiella pneumoniae* Bloodstream Infection From ST11 to ST15 at a Medical Center in Northeast China, 2015-2020, Infection and Drug Resistance. (2021) 14, 1855–1863, 10.2147/IDR.S311968, 34054300. 34054300 PMC8158045

[7] Pham M. H. , Hoi L. T. , Beale M. A. , Khokhar F. A. , Hoa N. T. , Musicha P. , Blackwell G. A. , Long H. B. , Huong D. T. , Binh N. G. , Co D. X. , Giang T. , Bui C. , Tran H. N. , Bryan J. , Herrick A. , Feltwell T. , Nadjm B. , Parkhill J. , van Doorn H. R. , Trung N. V. , van Kinh N. , Török M. E. , and Thomson N. R. , Evidence of Widespread Endemic Populations of Highly Multidrug Resistant *Klebsiella pneumoniae* in Hospital Settings in Hanoi, Vietnam: A Prospective Cohort Study, Lancet Microbe. (2023) 4, no. 4, e255–e263, 10.1016/S2666-5247(22)00338-X, 36801013. 36801013

[8] Wang M. , Earley M. , Chen L. , Hanson B. M. , Yu Y. , Liu Z. , Salcedo S. , Cober E. , Li L. , Kanj S. S. , Gao H. , Munita J. M. , Ordoñez K. , Weston G. , Satlin M. J. , Valderrama-Beltrán S. L. , Marimuthu K. , Stryjewski M. E. , Komarow L. , Luterbach C. , Marshall S. H. , Rudin S. D. , Manca C. , Paterson D. L. , Reyes J. , Villegas M. V. , Evans S. , Hill C. , Arias R. , Baum K. , Fries B. C. , Doi Y. , Patel R. , Kreiswirth B. N. , Bonomo R. A. , Chambers H. F. , Fowler V. G. , Arias C. A. , van Duin D. , Abbo L. M. , Anderson D. J. , Arias R. , Arias C. A. , Baum K. , Bonomo R. A. , Chambers H. F. , Chen L. , Chew K. L. , Cober E. , Cross H. R. , de P. P. , Desai S. , Dhar S. , di Castelnuovo V. , Diaz L. , Dinh A. N. Q. , Doi Y. , Earley M. , Eilertson B. , Evans B. , Evans S. , Fowler Jr V. G. , Fries B. C. , Gao H. , Garcia-Diaz J. , Garner O. B. , Greenwood-Quaintance K. , Hanson B. , Herc E. , Hill C. , Jacob J. T. , Jiang J. , Kalayjian R. C. , Kanj S. S. , Kaye K. S. , Kim A. , Komarow L. , Kreiswirth B. N. , Lauterbach C. , Li L. , Liu Z. , Manca C. , Marimuthu K. , Marshall S. H. , McCarty T. , Munita J. , Ng O. T. , Oñate Gutierrez J. M. , Ordoñez K. , Patel R. , Paterson D. L. , Peleg A. , Reyes J. , Rudin S. D. , Salata R. A. , Salcedo S. , Satlin M. J. , Schmidt-Malan S. , Smitasin N. , Spencer M. , Stryjewski M. , Su J. , Tambyah P. A. , Valderrama S. , van Duin D. , Villegas Botero M. V. , Wang M. , Waters M. , Weston G. , Wong D. , Wortmann G. , Yang Y. , Yu Y. , and Zhang F. , Clinical Outcomes and Bacterial Characteristics of Carbapenem-Resistant *Klebsiella pneumoniae* Complex Among Patients From Different Global Regions (CRACKLE-2): A Prospective, Multicentre, Cohort Study, Lancet Infectious Diseases. (2022) 22, no. 3, 401–412, 10.1016/S1473-3099(21)00399-6, 34767753. 34767753 PMC8882129

[9] Liu C. , Du P. , Xiao N. , Ji F. , Russo T. A. , and Guo J. , Hypervirulent *Klebsiella pneumoniae* is Emerging as an Increasingly Prevalent *K. pneumoniae* Pathotype Responsible for Nosocomial and Healthcare-Associated Infections in Beijing, China, Virulence. (2020) 11, no. 1, 1215–1224, 10.1080/21505594.2020.1809322, 32921250. 32921250 PMC7549996

[10] Tang H. L. , Chiang M. K. , Liou W. J. , Chen Y. T. , Peng H. L. , Chiou C. S. , Liu K. S. , Lu M. C. , Tung K. C. , and Lai Y. C. , Correlation Between *Klebsiella pneumoniae* Carrying pLVPK-Derived Loci and Abscess Formation, European Journal of Clinical Microbiology & Infectious Diseases. (2010) 29, no. 6, 689–698, 10.1007/s10096-010-0915-1, 2-s2.0-77952879122, 20383552. 20383552

[11] Yang X. , Xie M. , Xu Q. , Ye L. , Yang C. , Dong N. , Chan E. W. C. , Zhang R. , and Chen S. , Transmission of pLVPK-Like Virulence Plasmid in *Klebsiella pneumoniae* Mediated by an Incl 1 Conjugative Helper Plasmid, iScience. (2022) 25, no. 6, 104428, 10.1016/j.isci.2022.104428, 35663037. 35663037 PMC9160755

[12] Li P. , Liang Q. , Liu W. , Zheng B. , Liu L. , Wang W. , Xu Z. , Huang M. , and Feng Y. , Convergence of Carbapenem Resistance and Hypervirulence in a Highly-Transmissible ST11 Clone of *K. pneumoniae*: An Epidemiological, Genomic and Functional Study, Virulence. (2021) 12, no. 1, 377–388, 10.1080/21505594.2020.1867468, 33356821. 33356821 PMC7834077

[13] Yang X. , Wai-Chi Chan E. , Zhang R. , and Chen S. , A Conjugative Plasmid That Augments Virulence in *Klebsiella pneumoniae* , Nature Microbiology. (2019) 4, no. 12, 2039–2043, 10.1038/s41564-019-0566-7, 31570866. 31570866

[14] Lam M. M. C. , Wick R. R. , Watts S. C. , Cerdeira L. T. , Wyres K. L. , and Holt K. E. , A Genomic Surveillance Framework and Genotyping Tool for *Klebsiella pneumoniae* and Its Related Species Complex, Nature Communications. (2021) 12, no. 1, 10.1038/s41467-021-24448-3, 34234121. PMC8263825 34234121

[15] Seemann T. , Prokka: Rapid Prokaryotic Genome Annotation, Bioinformatics. (2014) 30, no. 14, 2068–2069, 10.1093/bioinformatics/btu153, 2-s2.0-84901417347, 24642063. 24642063

[16] Brown C. L. , Mullet J. , Hindi F. , Stoll J. E. , Gupta S. , Choi M. , Keenum I. , Vikesland P. , Pruden A. , and Zhang L. , mobileOG-db: A Manually Curated Database of Protein Families Mediating the Life Cycle of Bacterial Mobile Genetic Elements, Applied and Environmental Microbiology. (2022) 88, no. 18, e0099122, 10.1128/aem.00991-22, 36036594. 36036594 PMC9499024

[17] Sullivan M. J. , Petty N. K. , and Beatson S. A. , Easyfig: A Genome Comparison Visualizer, Bioinformatics. (2011) 27, no. 7, 1009–1010, 10.1093/bioinformatics/btr039, 2-s2.0-79953306448, 21278367. 21278367 PMC3065679

[18] Page A. J. , Cummins C. A. , Hunt M. , Wong V. K. , Reuter S. , Holden M. T. G. , Fookes M. , Falush D. , Keane J. A. , and Parkhill J. , Roary: Rapid Large-Scale Prokaryote Pan Genome Analysis, Bioinformatics. (2015) 31, no. 22, 3691–3693, 10.1093/bioinformatics/btv421, 2-s2.0-84947793096, 26198102. 26198102 PMC4817141

[19] Liu C. , Guo J. , Fan S. , Guo W. , Qi H. , Baker S. , du P. , and Cao B. , An Increased Prevalence of Carbapenem-Resistant Hypervirulent *Klebsiella pneumoniae* Associated With the COVID-19 Pandemic, Drug Resistance Updates. (2024) 77, 101124, 10.1016/j.drup.2024.101124, 39128195. 39128195

[20] Liu B. , Zheng D. , Zhou S. , Chen L. , and Yang J. , VFDB 2022: A General Classification Scheme for Bacterial Virulence Factors, Nucleic Acids Research. (2022) 50, no. D1, D912–D917, 10.1093/nar/gkab1107, 34850947. 34850947 PMC8728188

[21] Altschul S. F. , Gish W. , Miller W. , Myers E. W. , and Lipman D. J. , Basic Local Alignment Search Tool, Journal of Molecular Biology. (1990) 215, no. 3, 403–410, 10.1016/S0022-2836(05)80360-2, 2-s2.0-0025183708. 2231712

[22] Carattoli A. , Zankari E. , Garcia-Fernandez A. , Voldby Larsen M. , Lund O. , Villa L. , Møller Aarestrup F. , and Hasman H. , In Silico Detection and Typing of Plasmids Using PlasmidFinder and Plasmid Multilocus Sequence Typing, Antimicrobial Agents and Chemotherapy. (2014) 58, no. 7, 3895–3903, 10.1128/AAC.02412-14, 2-s2.0-84903160552, 24777092. 24777092 PMC4068535

[23] Jain C. , Rodriguez R. L. , Phillippy A. M. , Konstantinidis K. T. , and Aluru S. , High Throughput ANI Analysis of 90K Prokaryotic Genomes Reveals Clear Species Boundaries, Nature Communications. (2018) 9, no. 1, 10.1038/s41467-018-07641-9, 2-s2.0-85057599070, 30504855. PMC6269478 30504855

[24] Francisco A. P. , Vaz C. , Monteiro P. T. , Melo-Cristino J. , Ramirez M. , and Carriço J. A. , PHYLOViZ: Phylogenetic Inference and Data Visualization for Sequence Based Typing Methods, BMC Bioinformatics. (2012) 13, no. 1, 10.1186/1471-2105-13-87, 2-s2.0-84865286374. PMC3403920 22568821

[25] Zhang F. , Li L. , Zhao Y. , Dong H. , Zhao B. , Zhao X. , Xia Z. , Chi L. , Wang Y. , Li R. , Qin S. , and Fu X. , Molecular Characterization of Hybrid Virulence Plasmids in ST11-KL64 KPC-2-Producing Multidrug-Resistant Hypervirulent *Klebsiella pneumoniae* From China, Frontiers in Microbiology. (2024) 15, 1353849, 10.3389/fmicb.2024.1353849, 38550871. 38550871 PMC10972857

[26] Zhao H. , He Z. , Li Y. , and Sun B. , Epidemiology of Carbapenem-Resistant *Klebsiella pneumoniae* ST15 of Producing KPC-2, SHV-106 and CTX-M-15 in Anhui, China, BMC Microbiology. (2022) 22, no. 1, 10.1186/s12866-022-02672-1, 36319965. PMC9624029 36319965

[27] Lam M. M. C. , Wyres K. L. , Judd L. M. , Wick R. R. , Jenney A. , Brisse S. , and Holt K. E. , Tracking Key Virulence Loci Encoding Aerobactin and Salmochelin Siderophore Synthesis in *Klebsiella pneumoniae* , Genome Medicine. (2018) 10, no. 1, 10.1186/s13073-018-0587-5, 2-s2.0-85055612424, 30371343. PMC6205773 30371343

