# Supplementary material for: Genomic Evolution of the Increasing Prevalent Carbapenem‐Resistant Hypervirulent ST15 Klebsiella pneumoniae
Source: Int J Microbiol. 2026 May 8;2026:8275904. doi: 10.1155/ijm/8275904 (PMC13156470; doi:10.1155/ijm/8275904)
Supplement: Supplementary file 1 — Supporting Information 1 Figure S1: Gene sharing network of all (a) plasmids, and (b, c) highly virulent plasmids. The only distinction between (b) and (c) is the color of the interaction lines. [file IJM-2026-8275904-s003.docx]

a
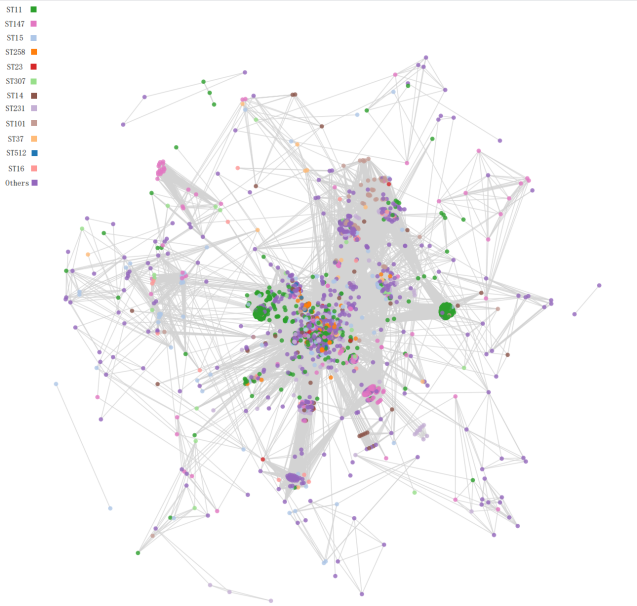
 b
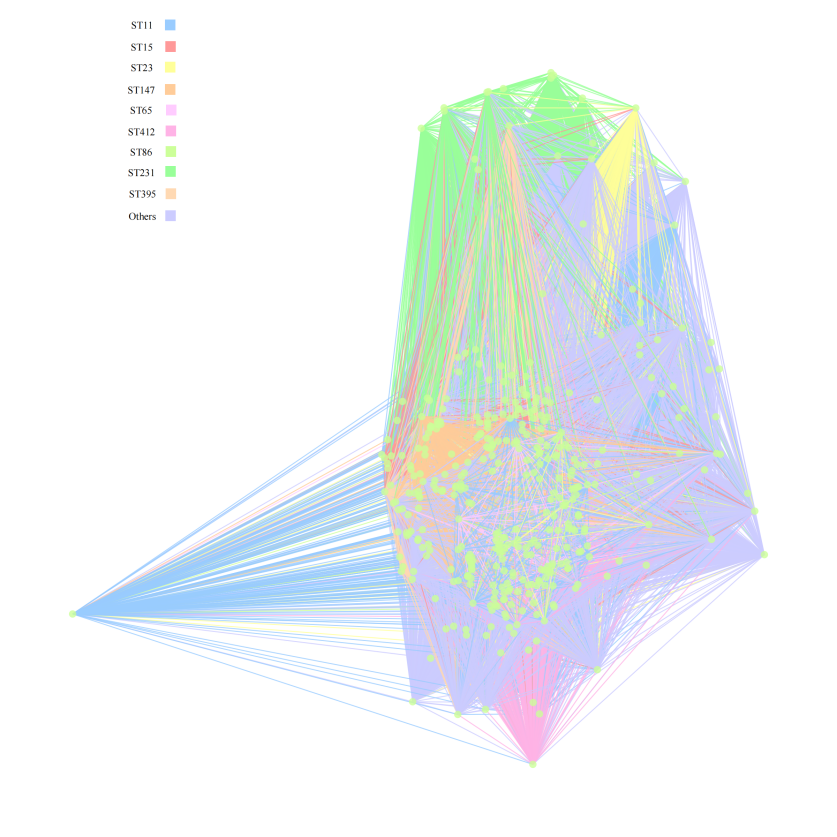
 c
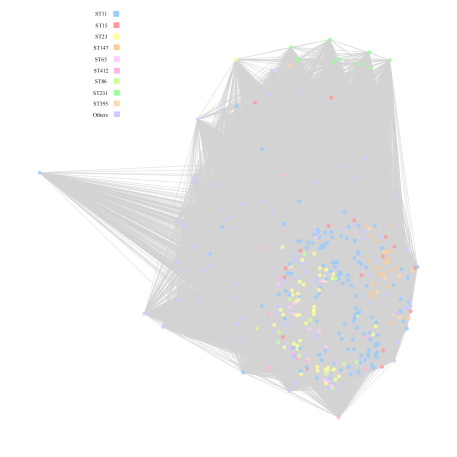


**Supplementary figure 1.** Gene sharing network of all plasmids (a), and highly virulent plasmids (b, c). The only distinction between (b) and (c) is the color of the interaction lines.
