# Supplementary material for: Genomic Evolution of the Increasing Prevalent Carbapenem‐Resistant Hypervirulent ST15 Klebsiella pneumoniae
Source: Int J Microbiol. 2026 May 8;2026:8275904. doi: 10.1155/ijm/8275904 (PMC13156470; doi:10.1155/ijm/8275904)
Supplement: Supplementary file 2 — Supporting Information 2 Figure S2: Detailed genomic locations of Figure 4: Collinearity analysis of the following plasmids with the pVir‐CR‐HvKp4 plasmid: (a) plasmid unnamed1 from Strain KP_NORM_BLD_2014_104014 (Accession Number CGF_003855315.1), (b) pKpvST15 from Strain KpvST15_NDM (Accession Number GCF_005885775.1), (c) p51015 NDM_1 from Strain 51015 (Accession Number GCF_011769825.1), and (d) Plasmid pHSKP1‐2 from Strain HSKP1 (Accession Number GCF_025884255.1). Additionally, we performed (e) a collinearity analysis between Plasmid pGZKP13‐1 (from Strain GZKP13; Accession Number GCF_025884275.1) and the pVir‐CR‐HvKp4 plasmid (derived from pHSKP1‐2). [file IJM-2026-8275904-s002.docx]

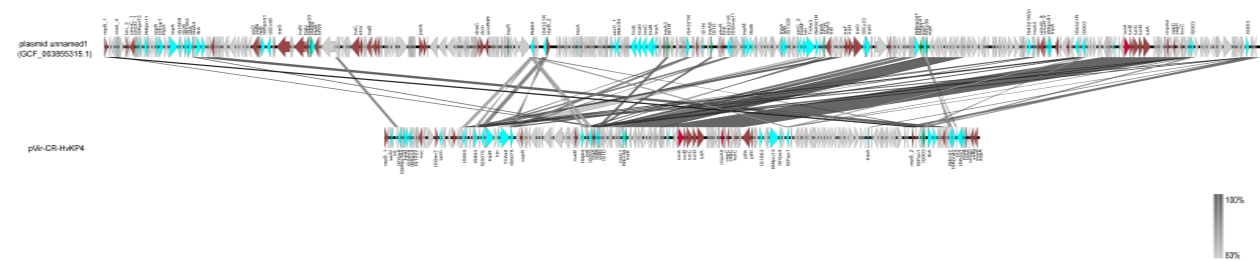

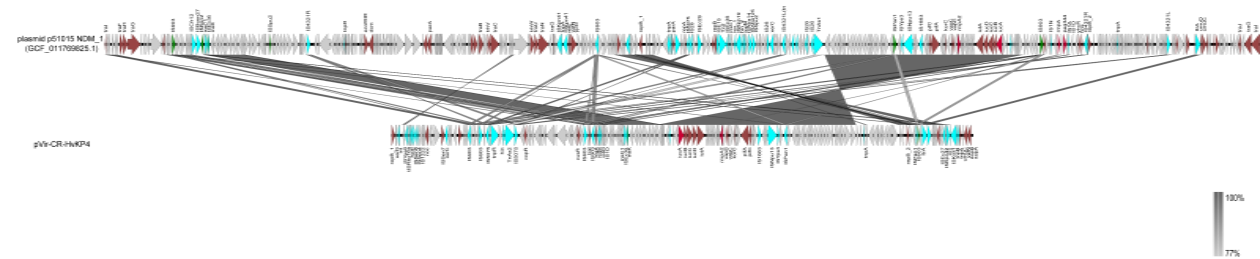

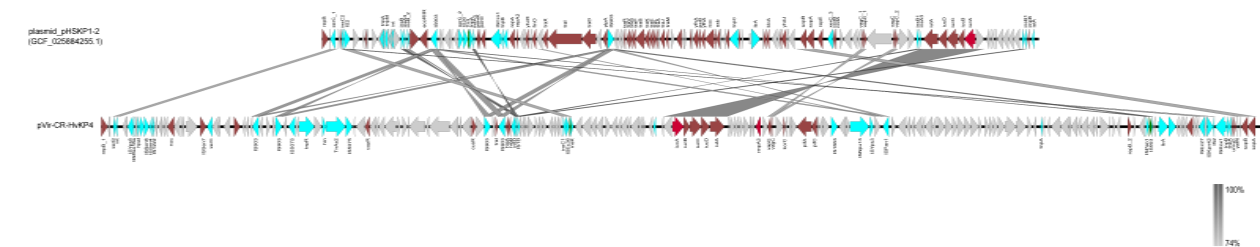

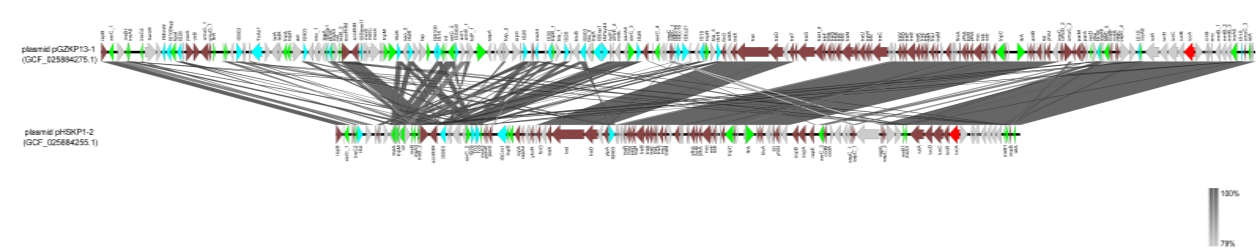


**Supplementary figure 2.** Detailed genomic locations of Fiure 4. Collinearity analysis of the following plasmids with the pVir-CR-HvKp4 plasmid:(a) plasmid unnamed1 from strain KP_NORM_BLD_2014_104014 (accession number: CGF_003855315.1), (b) pKpvST15 from strain KpvST15_NDM (accession number: GCF_005885775.1), (c) p51015 NDM_1 from strain 51015 (accession number: GCF_011769825.1), and (d) plasmid pHSKP1-2 from strain HSKP1 (accession number: GCF_025884255.1). Additionally, we performed a collinearity analysis between plasmid pGZKP13-1 (from strain GZKP13; accession number: GCF_025884275.1) and the pVir-CR-HvKp4 plasmid (derived from pHSKP1-2) (e).
