# Supplementary material for: Genomic Evolution of the Increasing Prevalent Carbapenem‐Resistant Hypervirulent ST15 Klebsiella pneumoniae
Source: Int J Microbiol. 2026 May 8;2026:8275904. doi: 10.1155/ijm/8275904 (PMC13156470; doi:10.1155/ijm/8275904)
Supplement: Supplementary file 3 — Supporting Information 3 Figure S3. Detailed genomic locations of Figure 6. Panel (a) shows the collinearity analysis of two virulence plasmids: pYSP8‐1‐CTX‐M‐14 and Plasmid pEH13_2 (GCF_021172125.1), compared with the ST15 Plasmid pHSKP1‐2 (GCF_025884255.1). Panel (b) focuses on the collinearity analysis of the same two virulence plasmids, pYSP8‐1‐CTX‐M‐14 and Plasmid pEH13_2. Panel (c) examines the collinearity analysis of two nonvirulence plasmids: 30348_1 Plasmid 2 and pGDD25‐5, in relation to the ST15 Plasmid pHSKP1‐2. [file IJM-2026-8275904-s004.docx]

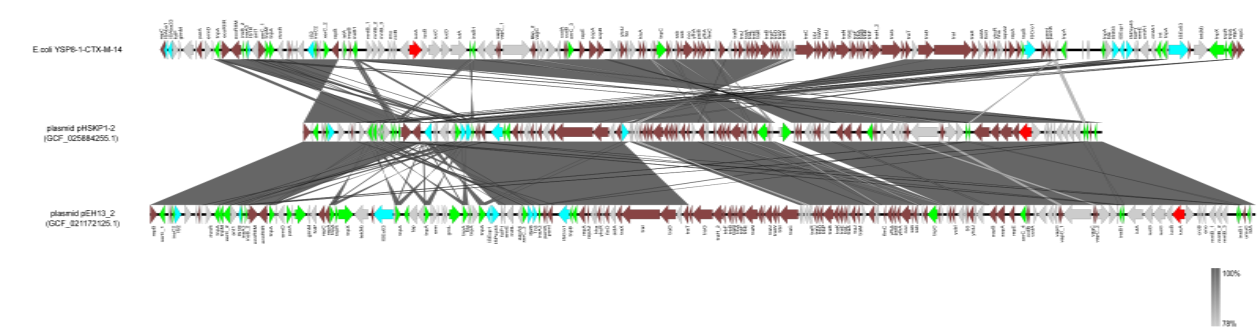

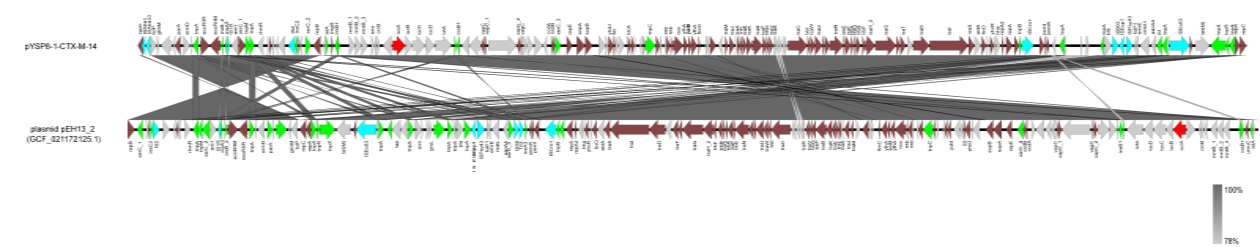

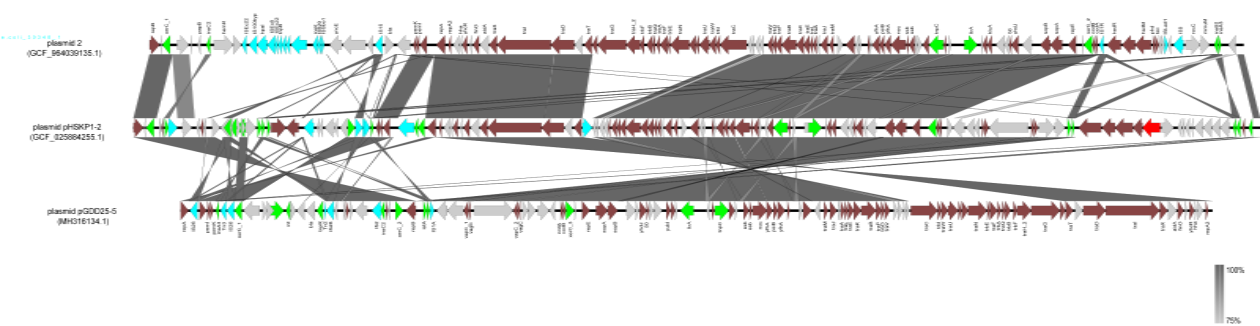


**Supplementary figure 3.** Detailed genomic locations of Fiure 6. Figure a shows the collinearity analysis of two virulence plasmids: pYSP8-1-CTX-M-14 and plasmid pEH13_2 (GCF_021172125.1), compared to the ST15 plasmid pHSKP1-2 (GCF_025884255.1). Figure b focuses on the collinearity analysis of the same two virulence plasmids, pYSP8-1-CTX-M-14 and plasmid pEH13_2. Figure c examines the collinearity analysis of two nonvirulence plasmids: 30348_1 plasmid 2 and pGDD25-5, in relation to the ST15 plasmid pHSKP1-2.
