# Supplementary material for: Genomic Evolution of the Increasing Prevalent Carbapenem‐Resistant Hypervirulent ST15 Klebsiella pneumoniae
Source: Int J Microbiol. 2026 May 8;2026:8275904. doi: 10.1155/ijm/8275904 (PMC13156470; doi:10.1155/ijm/8275904)
Supplement: Supplementary file 4 — Supporting Information 4 Figure S4. Detailed genomic locations of Figure 7b. Collinearity analysis between plasmid p1 and pVir‐CR‐HvKp4. [file IJM-2026-8275904-s006.docx]

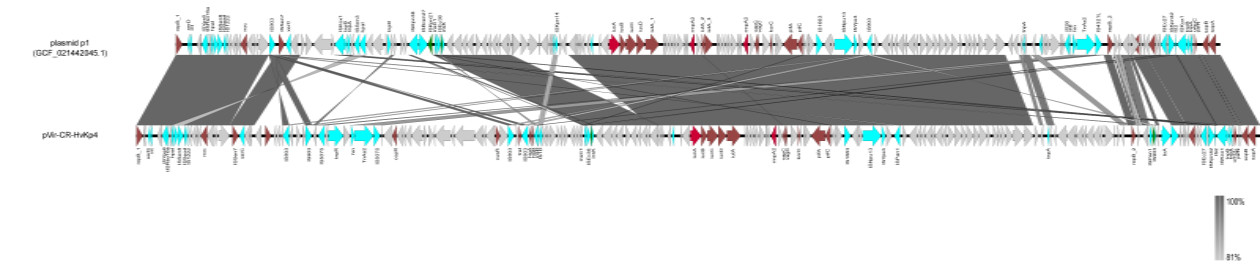


**Supplementary figure 4.** Detailed genomic locations of Fiure 7b. Collinearity analysis between plasmid p1 and pVir-CR-HvKp4.
