# Supplementary material for: Genomic Evolution of the Increasing Prevalent Carbapenem‐Resistant Hypervirulent ST15 Klebsiella pneumoniae
Source: Int J Microbiol. 2026 May 8;2026:8275904. doi: 10.1155/ijm/8275904 (PMC13156470; doi:10.1155/ijm/8275904)
Supplement: Supplementary file 5 — Supporting Information 5 Table S1: This table provides a detailed breakdown of the virulence gene repertoire for all 17 ST15 hv‐CRKP isolates analyzed in this study, listing the specific loci present in each isolate. [file IJM-2026-8275904-s005.docx]

Supplementary tab. 1

| strain | *iucA* | *rmpA2* | *rmpA2*_sec | *peg344* |
| --- | --- | --- | --- | --- |
| GCF_003855315.1_plasmid_unnamed1 | 305140:306864 | 317997:318631 |  |  |
| GCF_005885775.1_plasmid__pKpvST15 | 8993:10717 | 22178:22816 |  |  |
| GCF_009884415.1_plasmid_pWSD411_2 | 89390:91114 | 102248:102886 |  |  |
| GCF_011769825.1_plasmid_p51015_NDM_1 | 273075:274799 | 261301:261940 |  | 293600:294502 |
| GCF_019334545.1_plasmid_pKP3295-1 | 89390:91114 | 102248:102886 |  |  |
| GCF_021442045.1_plasmid_p1 | 68659:70383 | 89996:90634 | 81517:82155 |  |
| GCF_023066625.1_plasmid_pDD02172-1 | 101735:103459 | 114593:115228 |  |  |
| GCF_024637995.1_plasmid_pKPTCM-1 | 78850:80574 | 91708:92346 |  |  |
| GCF_025884095.1_plasmid_pRJKP36-2 | 166918:168642 | 155146:155784 |  |  |
| GCF_025884255.1_plasmid_pHSKP1-2 | 98716:100440 |  |  |  |
| GCF_025884275.1_plasmid_pGZKP13-1 | 174479:176203 |  |  |  |
| GCF_025946965.1_plasmid_pKP424-2 | 103428:105152 | 116286:116921 |  |  |
| GCF_030489785.1_plasmid_p2016N17-469-265k | 160889:162613 | 173747:174381 |  |  |
| GCF_030845575.1_plasmid_p1 | 93648:95372 | 106506:107144 |  |  |
| GCF_030845595.1_plasmid_p1 | 93649:95373 | 106507:107145 |  |  |
| GCF_030845615.1_plasmid_p1 | 93649:95373 | 106507:107145 |  |  |
| GCF_030845815.1_plasmid_p2 | 117484:119208 | 130342:130976 |  | 94326:95228 |
