# Supplementary material for: Genomic Evolution of the Increasing Prevalent Carbapenem‐Resistant Hypervirulent ST15 Klebsiella pneumoniae
Source: Int J Microbiol. 2026 May 8;2026:8275904. doi: 10.1155/ijm/8275904 (PMC13156470; doi:10.1155/ijm/8275904)
Supplement: Supplementary file 6 — Supporting Information 6 This Supporting Information analyzes temporal trends in the resistance and virulence of clinical K. pneumoniae, revealing a significant global increase in CRKP since 2010. [file IJM-2026-8275904-s001.docx]

**The clinical strains of ST15 Kp is increasing**

The genomes of the 14,728 clinical isolates of Kp from NCBI originated from Asia (6,409), Europe (4,536), North America (1,900), Africa (1,026), South America (590), and Oceania (267). These Kp isolates were categorized into 1070 different STs. The 10 most common STs accounted for 54.3% (7,990/14,728) of the selected genomes, whereas the top five STs represented 40.6% of all sequences. The top five STs were ST11 (2,609, 17.7%), ST258 (1,191, 8.1%), ST15 (974, 6.6%), ST147 (656, 4.5%), and ST307 (553, 3.8%), and these isolates were primarily from Asia, Europe, and North America. Specifically, ST15 isolates were mainly from Asia (62.8%) and Europe (26.2%).

a
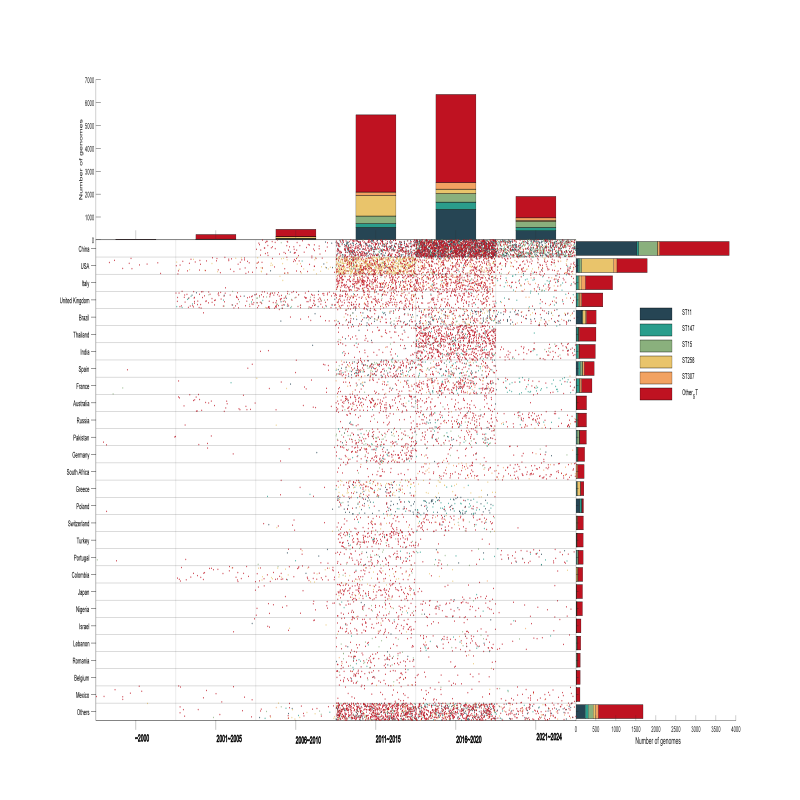


b
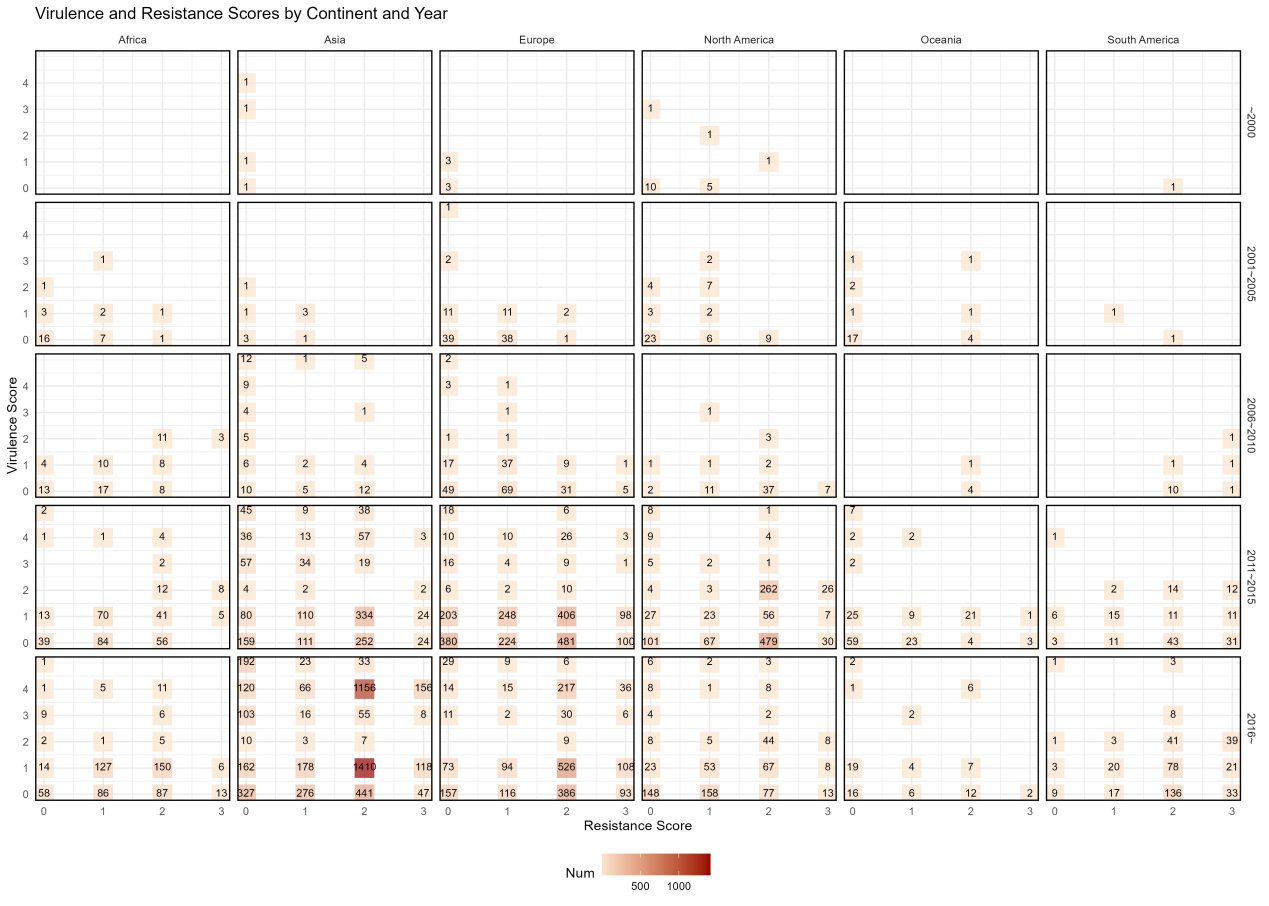


c
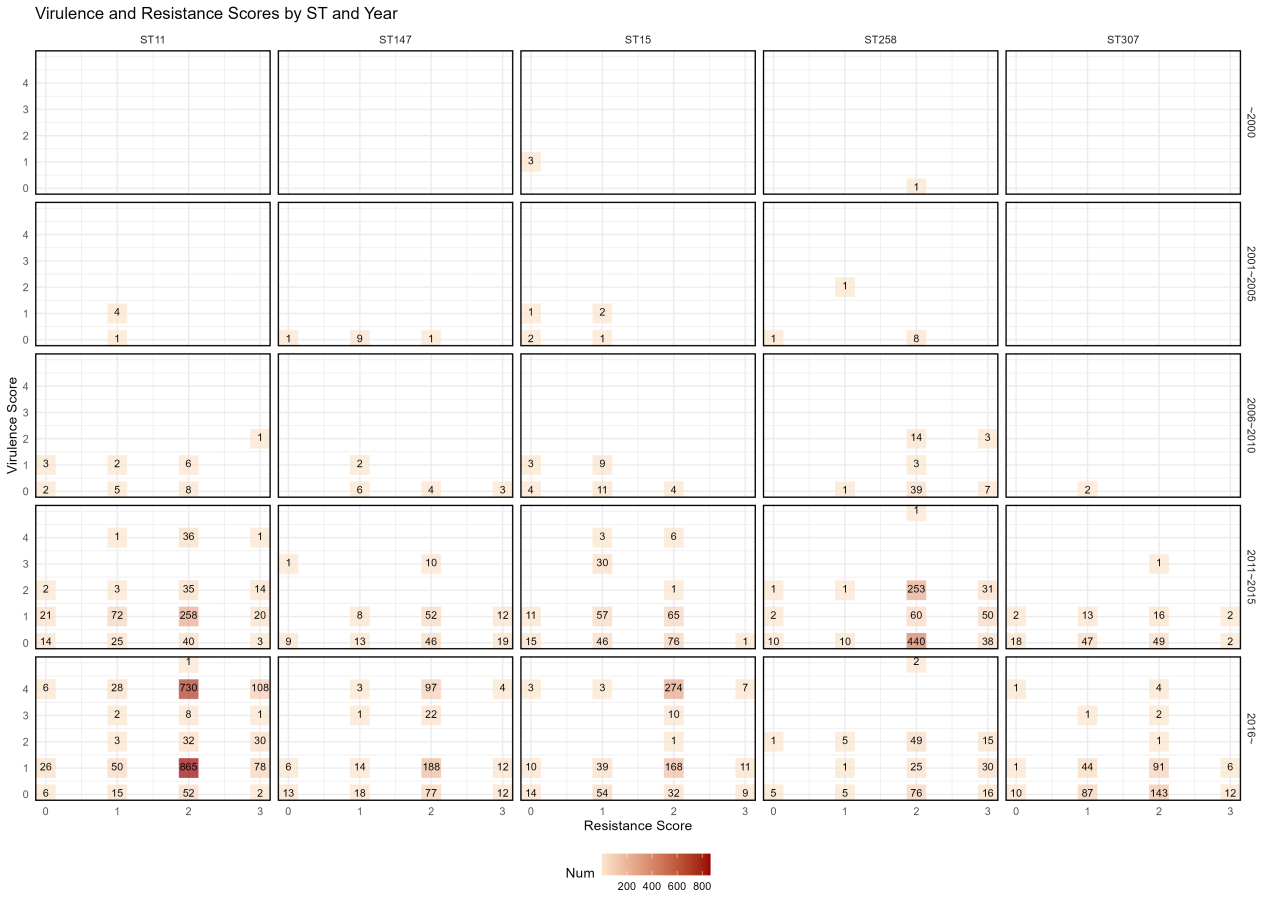


The resistance and virulence of Kp have significantly increased over time across different continents (Fig. b). Notably, since 2010, CRKP has shown a significant upward trend, particularly in ST11 and ST15, with the proportion of ST15 increasing markedly in Asia, especially in China (Fig. a, c).
